# Acquired bronchoesophageal fistula

**DOI:** 10.4103/0970-2113.45201

**Published:** 2009

**Authors:** Deepak Aggarwal, Prasanta Raghab Mohapatra, Balbir Malhotra

**Affiliations:** *Department of Pulmonary Medicine, Government Medical College and Hospital, Chandigarh, India*; 1 *Department of Tuberculosis and Respiratory Diseases, Government Medical College, Amritsar, India*

**Keywords:** Bronchoesophageal fistula, endoscopy, dysphagia, acquired

## Abstract

Bronchoesophageal fistula in an adult is rarely encountered in clinical practice. Most commonly, they have malignant origin. We report a case of bronchoesophageal fistula secondary to trauma caused by upper gastrointestinal endoscopy. The patient presented with recurrent chest infections and dysphagia since he underwent endoscopic procedure for obstructed denture. Barium swallow study revealed fistulous connection between right lower lobe bronchus and esophagus.

## INTRODUCTION

Bronchoesophageal (BE) fistula, as the name suggests, is a communication between bronchus and esophagus. It may be congenital or acquired. Congenital BE fistula has been reported to be 25–50% less common than tracheoesophageal fistula,^1^ while the incidence of acquired BE fistula is not known. Acquired causes of BE fistula include malignancies, infections, and traumatic factors like prolonged endotracheal intubation^2^ and blunt chest injury.^3^ Esophageal foreign bodies resulting in tracheoesophageal fistula have been reported,^4^ but association between endoscopy/esophageal foreign body and BE fistula has been scarcely documented in literature.^5^

## CASE HISTORY

A 40-year-old male presented in our OPD with cough, fever, and dysphagia of 2 weeks duration. Cough was accompanied by mucopurulent, nonfoul smelling expectoration, about 30–40 ml/day. The patient had similar complaints for last three years, which started soon after he underwent upper gastrointestinal endoscopy for obstructed denture in the esophagus. Since then, there had been no improvement with antibiotics and symptomatic treatment.

**Figure 1:**
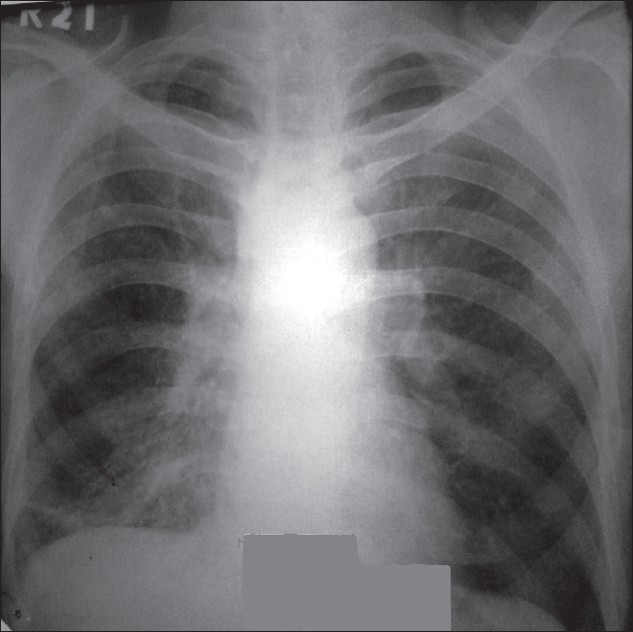
Chest radiograph shows right lower zone heterogenous shadows

**Figure 2:**
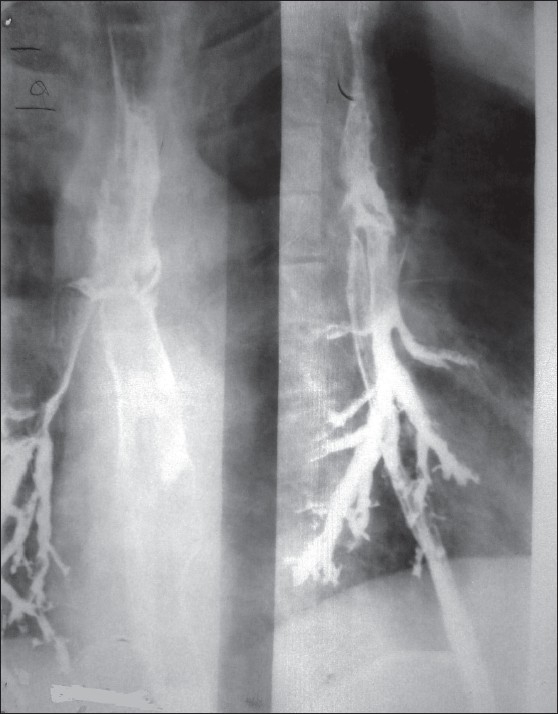
Barium swallow study showing barium outlining esophagus, fistulous tract, and the right lower lobe bronchus

On general examination, the patient was poorly nourished. Chest examination revealed infrascapular rales on the right side. Examination of other systems was unremarkable. X-ray chest showed lower zone heterogenous shadows on the right side [Figure 1] while sputum microscopy was negative for acid fast bacilli. His hemogram, blood sugar, renal, and liver function tests were all within normal limits. To evaluate dysphagia, barium swallow study was done. It revealed barium (contrast) outlining the esophagus, fistulous tract, and the right lower lobe bronchus [Figure 2].

## DISCUSSION

BE fistulae in adults are less commonly reported in literature. Congenital BE fistulae are usually diagnosed in neonatal period, but in few cases, they may remain silent till adulthood.^6^ Adult BE fistulae are mostly acquired in nature.^2^ Their exact incidence has not been reported in India.

Acquired causes of BE fistula include malignancies involving esophagus or adjacent structures. Benign conditions causing fistula are less common and consist of infections like tuberculosis, syphilis, histoplasmosis, actinomycosis, and candidiasis.^2^ Tuberculosis being endemic in India should always be considered in the differential diagnosis of BE fistula. Traumatic factors like prolonged endotracheal intubation 2 and blunt chest injury^3^ have also been associated with BE fistulae but endoscopic intervention or esophageal foreign body resulting in BE fistula has been rarely documented in literature.^5^,^7^ Other conditions known to cause fistula include inflammatory conditions like Crohn's disease and Behect's disease, broncholithiasis, and corrosive ingestion^2^.

Acquired fistulae are frequently misdiagnosed. They are characterized by bouts of coughing while eating or drinking (Ohno's sign) and with recurrent pulmonary infections.^2^ Delay in diagnosis may be complicated by pneumonia, life-threatening hemoptysis, and respiratory failure. Conventional barium esophagography is considered to be the most sensitive test for diagnosing BE fistula. This investigation provides a definitive diagnosis in 78% of cases.^2^

The acquired nature of the fistula can be proven by demonstrating the acquired cause and by the absence of normal mucosa lining the fistulous tract. The acquired nature of the fistula in our case may be assumed due to the presence of temporal relationship between the endoscopic intervention and the onset of symptoms soon after it. Both, foreign body (denture) and the therapeutic intervention (endoscopy) to treat it could be the cause of BE fistula in our case.

To conclude, acquired BE fistulae can have diverse and rare causes like endoscopic intervention or ingested foreign bodies. High index of suspicion is required to diagnose the fistula and ascertain its cause. The diagnosis should be considered while managing such patients especially when no other cause is evident.
